# Neglected tropical disease targets must include morbidity

**DOI:** 10.1016/S2214-109X(15)00185-0

**Published:** 2015-10

**Authors:** Kebede Deribe

**Affiliations:** Brighton & Sussex Medical School, Brighton, UK; and School of Public Health, Addis Ababa University, Addis Ababa, Ethiopia

The Royal Society of Tropical Medicine and Hygiene is holding a 1-day meeting in London, UK, on Sept 25, 2015, to discuss “The disease elimination agenda: the role of science, policy and advocacy”.^1^ Important meetings such as this are a welcome forum at which to discuss progress and challenges and to reflect on important milestones, particularly with regard to neglected tropical diseases (NTDs). Such NTD milestones include the London declaration^2^ and the WHO roadmap.^3^ However, the success of these elimination initiatives is contingent on inclusive programming and on addressing the problem in its entirety.

Most of the targets for elimination focus on interrupting transmission and infection cycles, yet many of the NTDs cause severe morbidity including disabling lymphoedema, massive hydrocele, disfigurement, and blindness.^4^ Despite the huge burden of morbidities, there are no clear targets towards their elimination, and, with the exception of trachoma, there are no morbidity indictors to measure the success of elimination.^5^ A more inclusive approach to addressing morbidity in the elimination of NTDs should focus on the following points.

First, elimination targets should clearly include indicators related to morbidity. Such indicators should go beyond measuring access to care and should bind success to the extent of morbidity alleviation. WHO’s trachoma elimination target of a prevalence of active trachoma of less than 5% among children aged 1–9 years and a prevalence of trachoma trichiasis of less than one case per 1000 population^5^ successfully combines both prevention of new infections and reduction of morbidity, and should be replicated across the different diseases.

Second, resources should be clearly committed to the morbidity management aspect of these NTDs. Funding such as the USAID’s support of Helen Keller International’s Morbidity Management and Disability Prevention for Blinding Trachoma and Lymphatic Filariasis Project is welcome.^6^ Given the scale of the problem, more resources to address the morbidity challenge are required. As resources are directed towards preventing new infection, equally resources should also be targeted to improving the quality of life of the people suffering from the consequences of the diseases.

Third, operational research into optimising the delivery of morbidity management services is also important. One of the challenges of scaling up such services is a dearth of evidence on how to integrate them into the existing health systems and ongoing NTD programmes. Operational research focusing on integration of services, surveillance, and barriers to the existing services will be important.

To achieve the challenges of elimination, morbidity management is essential, not optional. Strong advocacy and awareness-raising for donors is important, but the change should start from within by including morbidity targets in some of the NTD elimination targets.
